# A Study of the Demographic Profile, Screening, and Management of Patients Visiting a Retinopathy of Prematurity Clinic

**DOI:** 10.7759/cureus.58305

**Published:** 2024-04-15

**Authors:** Iqra Mushtaq, Tushar Agrawal, Deepaswi Bhavsar, Shaili Choudhary, Prachi N Bakare

**Affiliations:** 1 Ophthalmology, Dr. D. Y. Patil Medical College, Hospital & Research Centre, Pune, IND; 2 Ophthalmology, Post Graduate Institute, Yashwantrao Chavan Memorial Hospital, Pune, IND

**Keywords:** low birth weight, prematurity, demography, incidence, retinopathy of prematurity

## Abstract

Background and objective

Retinopathy of prematurity (ROP) is a retinal vasoproliferative disease affecting premature infants. Despite improvements in neonatal care and management, ROP still remains a major cause of childhood blindness worldwide. Studying the demographic profile and screening is essential to develop predictive models, to gain insights into the cause of retinal vascular diseases and diseases of prematurity, and to determine the future management and research in ROP.

The objective of the present study was to estimate the incidence of ROP, to identify the risk factors that predispose to ROP, and to assess the outcome of these cases. Hence, this study was conducted in a tertiary care hospital in Maharashtra.

Method

A prospective, observational study was conducted from 10 August 2022 to 10 October 2022. Infants with gestational ages < 34 weeks, birth weights < 2000 g, infants who received supplemental oxygen therapy, or patients who required NICU stay were screened for ROP. Demographic details were recorded to assess the risk factors and treatment was given according to the severity of ROP grade.

Result

A total of 160 eyes of 80 infants were screened and analysed. The overall incidence of “any ROP” was 19 patients (38 eyes), i.e., 24%. Out of 80 patients, six were of 28 weeks gestational age, of whom four (67%) were positive for ROP. The mean birth weight of infants with ROP was 1331.58 ± 238.532 g (p < 0.0001). ROP stage 1 was seen in five patients (26.32%), stage 2 in 10 patients (52.63%), and stage 3 in four patients (21.0%), with no subjects in stages 4 & 5. Out of 19 patients, six (32%) had type 1 ROP, and 13 (68%) had type 2 ROP. Out of 19 cases, 13 (68%) received follow-up care based on the severity of their disease, and six (32%) were treated with panretinal photocoagulation (PRP) laser.

Conclusion

Incidence of any ROP was 24%. Prematurity, low birth weight, and oxygen therapy remain the most significant risk factors associated with the development of ROP. Early referral, diagnosis, and timely intervention will play a monumental role in improving the prognosis of this potentially blinding disease.

## Introduction

The incidence of retinopathy of prematurity (ROP), a multifactorial vasoproliferative retinal condition, rises with a lower gestational age. In premature newborns, it is typified by the formation of aberrant immature blood vessels in the retina. These aberrant blood vessels can rupture and produce haemorrhage, which can result in retinal detachment or scarring. They are also brittle and friable.

Worldwide, 10% of babies are born prematurely [1]. Recent improvements in neonatal care have boosted the survival rates of preterm infants, which has led to a corresponding rise in the incidence of ROP. One of the main causes of avoidable childhood blindness is ROP. ROP is thought to impact about 50,000 newborns globally each year. It is estimated that each year, 500 youngsters in India lose their sight due to ROP [2]. India's rural areas have reported a high incidence of ROP over the past 10 years, ranging from 22.4% to 41.5%, while urban and semi-urban areas have recorded incidences ranging from 14.8% to 26.6% [3]. In both middle-class and high-income countries, ROP has been linked to childhood blindness, according to the World Health Organization's 1999 VISION 2020 plan. Childhood blindness is one of the five priority areas that the programme has designated [4].

Reduced gestational age, low birth weight, and prolonged exposure to extra oxygen after delivery are factors that have a persistent and significant correlation with reduced oxygen production. Additional risk factors include apnoea, sepsis, anaemia, surfactant therapy, mechanical breathing, vaginal delivery, and frequent blood transfusions. We still do not know exactly how these factors affect the onset and course of ROP [5]. ROP-related blindness can be prevented with a straightforward screening exam performed by an ophthalmologist a few weeks after birth. Screening should be performed on infants who are 34 weeks of gestational age or below, weigh 2000 grams or less at birth, or are older than 34 weeks of gestational age and have any associated risk factors. But in developing nations like India, screening protocols are not as stringently adhered to as they are in affluent nations. In India, expanding ROP screening and treatment services is necessary to prevent vision loss due to ROP. ROP is a dynamic, time-bound illness that requires extra care. The prognosis is greatly influenced by early diagnosis, prompt referral by a neonatologist, and awareness. Early detection of retinal harm and the implementation of suitable treatment can both offer the child improved development and prevent blindness.

Numerous investigations have previously been carried out to learn more about the demographics, screening, and treatment of individuals visiting the ROP clinic. Nevertheless, none of them have brought up the socioeconomic standing of the people who are coming in for screening. Since the prevalence of ROP is rising in India, it is clear that not everyone can access healthcare services, including screening and therapy for the condition. There could be a number of reasons for that, including ignorance and incomplete knowledge. In this study, we have analysed a new variable, namely, the socioeconomic status of the newborns' family, to determine whether socioeconomic status is another reason for inadequate screening.

## Materials and methods

Study overview

A prospective, observational study was carried out at a tertiary care centre in Maharashtra between 10 August 2022 and 10 October 2022.

Ethical considerations

The project was approved by the Institutional Ethics Sub-Committee, Dr. D.Y. Patil Medical College (Approval No.: I.E.S.C./106/2022).

Study criteria

Study criteria included infants of gestational age of 34 weeks or less, birth weight of 2000 grams or less, infants who received supplemental oxygen therapy, or who required NICU stay.

The infants meeting the inclusion criteria (at least one) were recruited prospectively from the same period and their weight, gestational age, gender, age at presentation, and the socioeconomic status of the family were recorded. Patients who were lost to follow-up were excluded from the study.

Procedure

Before screening and definitive examination, the study was explained in detail to all the participants (parents). Written consent was obtained from all the participants (in this case, the parents of the participating infants). The demographic data and detailed clinical history were obtained from the parents with a special note taken on oxygen administration; socioeconomic status was written according to the modified Kuppuswamy scale. Documentation was done in a proforma.

A fixed-dose combination of tropicamide 0.5% and phenylephrine 2.5% was used for the dilation of pupils 20 minutes before the examination. Topical anaesthetic drops (proparacaine hydrochloride 0.5%) were instilled and a paediatric wire speculum was applied. Fundus examination was done using a binocular indirect ophthalmoscope with a 20 D or a 28 D lens. All the examinations were performed by an ROP expert. The details of ROP at the presentation were documented in terms of zone, stage, and the presence or absence of plus disease in accordance with the current International Classification of Retinopathy of Prematurity (ICROP) classification. Infants with normal complete vascularization of the fundus up to the periphery were not re-examined. Those with peripheral avascular retina but no clinical ROP were re-examined at weekly intervals.

Assessments

The diagnosed cases of ROP were classified as type 1 and type 2 according to the ICROP 2021 classification. Infants falling in the type 1 category were given treatment with panretinal photocoagulation (PRP) laser within 72 hours of diagnosis. Follow-up post-laser was done at an interval of three to seven days to access the anatomical result. In cases where adequate signs of regression were not observed, a second laser sitting was added to the treatment. Post-treatment examinations were continued for the entire duration of our study and were continued further till four years of age. The infants falling in the type 2 category were called for follow-up based on the severity of their disease. They were observed carefully to watch for signs of regression or progression needing treatment. For our study purpose, the final visit was till the retina was completely vascularized or ROP was regressed, but outside the study, follow-up was continued till one year of age.

Sample size calculation

Considering a confidence interval of 95% and 80% power, the sample size included for our study was 160 eyes of 80 infants by WinPepi version 11.38 software.

Statistical analysis

Follow-up of the patients in one week or less was classified into zone I ROP (stage 1 or 2) or zone II ROP (stage 3), while one to two weeks were classified into zone I regressing ROP, zone I with immature vasculature, or zone II ROP (stage 2); patients with a follow-up in two weeks were classified into zone II regressing ROP or zone II ROP (stage 1), while those with a follow-up in two to three weeks were classified into zone II ROP with immature vasculature or zone III regressing ROP.

The type of treatment received by each baby (follow-up or PRP laser) and the number of laser sittings were documented. Data were entered in Microsoft Excel (Microsoft Corporation, Redmond, WA) and all the statistical analyses were conducted using Epi Info software version 5.5.9 (Centers for Disease Control and Prevention, Atlanta, GA). Descriptive statistical methods were used to describe the study sample. Qualitative data were represented in the form of frequencies and percentages. Quantitative data were summarized using mean, median, and standard deviation and an unpaired t-test was used wherever required.

Comparison for categorical variables was performed using the chi-square test. Fisher’s exact test was used when the expected cell count was less than 5 and the chi-square was not valid. The results were considered statistically significant at p-value < 0.05, with 95% confidence intervals (CI). The odds ratio (95% CI) was obtained for the categorical variables, which has been shown to be significant by the chi-square test or Fisher’s exact test.

## Results

A total of 160 eyes of 80 subjects were examined in this study, which included infants of birth weight of 2000 grams or less, gestational age of 34 weeks or less, infants who received supplemental oxygen therapy, or those who required NICU stay.

Incidence of ROP in the study

**Figure 1:**
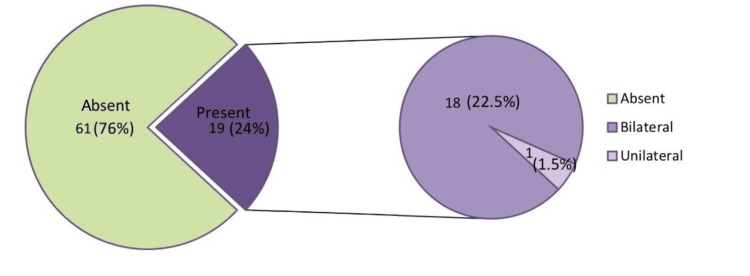
Incidence of retinopathy of prematurity

Figure 1 shows that out of 80 babies, 19 developed ROP and 61 did not develop ROP. So, the incidence of ROP in our study was 24% and out of the ROP-positive infants (19, 24%), 18 (22.5%) developed bilateral ROP whereas one (1.5%) developed unilateral ROP.

Gestational age at the time of birth and ROP

**Figure 2:**
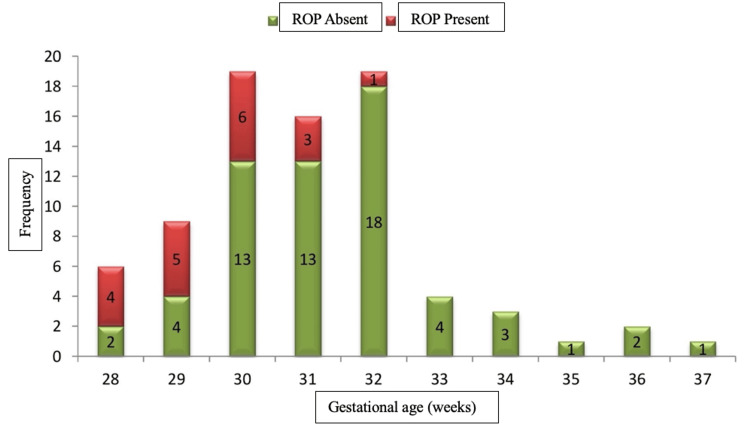
Gestational age and ROP ROP: retinopathy of prematurity.

Figure 2 shows the total number of subjects in each gestational age (at birth) and the number of infants who were ROP positive and ROP negative.

In our study of 80 infants, six were 28 weeks of gestational age (at the time of birth), out of whom four (67%) were ROP positive and two (33%) were ROP negative. Nine babies were of gestational age of 29 weeks, out of whom five (56%) were ROP positive and four (44%) were ROP negative. Nineteen babies were of gestational age of 30 weeks, out of whom six (32%) were ROP positive and 13 (68%) were ROP negative. Sixteen babies were of gestational age of 31 weeks, out of whom three (19%) were ROP positive and 13 (81%) were ROP negative. Nineteen babies were of gestational age of 32 weeks, out of whom one (5%) was ROP positive and 18 (95%) were ROP negative. Four babies were of gestational age of 33 weeks, three were of 34 weeks, one was of 35 weeks, two were of 36 weeks, and one was of gestational age of 37 weeks. All of these babies were ROP-negative.

The mean gestational age of infants in our study of 80 subjects was 31.01 ± 1.859 weeks, with a maximum of 37 weeks and a minimum of 28 weeks.

Table 1 shows the mean gestational age of infants with ROP and without ROP, i.e., 29.58 ± 1.139 weeks and 31.46 ± 1.179 weeks, respectively. The difference between them was found statistically significant on the application of a t-test with p < 0.0001 and t = 6.1166.

**Table 1:** Distribution of infants based on gestational age and incidence of ROP ROP: retinopathy of prematurity.

ROP	N	Gestational age (weeks)		
		Mean ± SD	Minimum	Maximum
Present	19	29.58 ± 1.139	28	32
Absent	61	31.46 ± 1.179	28	37

Distribution of birth weight among study subjects

Table 2 shows the birth weight distribution of study subjects and the number of infants with and without ROP in each birth weight category.

**Table 2:** Distribution of birth weight among study subjects ROP: retinopathy of prematurity.

Birth weight (grams)	Total	ROP present	ROP absent
<=1000	3	3	0
1001-1250	8	5	3
1251-1500	25	7	18
1501-1750	17	4	13
1751-2000	19	0	19
>2000	8	0	8
Total	80	19	61

In our study, the mean birth weight of infants was 1618 ± 339.0 grams, with a maximum of 2500 grams and a minimum of 900 grams.

Table 3 shows the mean birth weight of infants with ROP and without ROP, i.e., 1331.58 ± 238.532 g and 1706.56 ± 315.641 g, respectively. The difference between them was found statistically significant on the application of a t-test with p < 0.0001 and t = 4.7637.

**Table 3:** Distribution of infants based on birth weight and incidence of ROP ROP: retinopathy of prematurity.

ROP	N	Birth weight (grams)		
		Mean ± SD	Minimum	Maximum
Present	19	1331.58 ± 238.532	900	1700
Absent	61	1706.56 ± 315.641	1100	2500

Age at the time of first screening

The mean age of infants in our study was 2.76 ± 2.225 grams, with a maximum of 10 months and a minimum of 0.5 months.

Socioeconomic status of the study subjects

**Figure 3:**
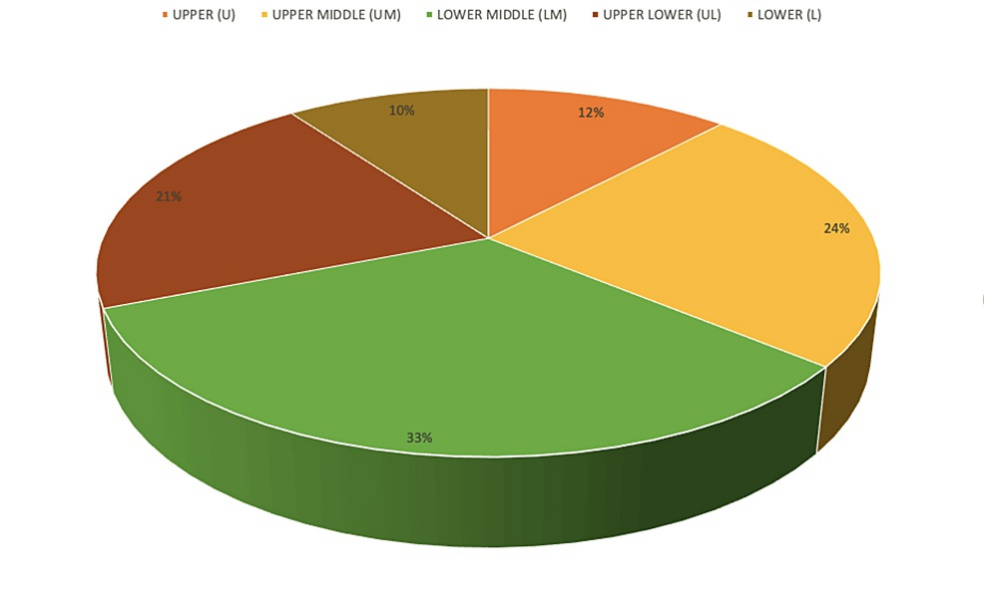
Socioeconomic status of study subjects

Figure 3 shows a pie chart with 12% of our subjects belonging to upper-class families, 24% belonging to upper-middle-class families, 33% belonging to lower-middle-class families, 21% belonging to upper-lower-class families, and 10% of the subjects belonging to lower-class families.

Gender distribution of the study subjects

**Figure 4:**
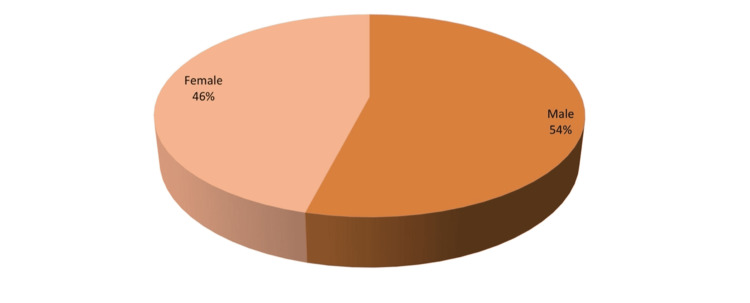
Gender distribution of study subjects

Figure 4 shows a pie chart of 80 subjects with 37 (46%) females and 43 (54%) males.

Association between ROP and gender

Table 4 shows that out of 19 babies with ROP, 10 (52.6%) were males and nine (47.4%) were females. A chi-square test of independence was performed to examine the association between ROP and gender. Statistical analysis was done.

**Table 4:** Association between ROP and gender ROP: retinopathy of prematurity.

Gender	ROP		
	Present	Absent	Total
Female	9	28	37
Male	10	33	43
Total	19	61	80

The chi-square statistic was 0.012582 with a degree of freedom of 1, and the p-value came out to be 0.910844. So, there was no association found between ROP and gender as the p-value was >0.05 and hence the result was insignificant.

Association between ROP and oxygen supplementation therapy

Table 5 shows that out of 19 subjects diagnosed with ROP in this study, 16 (84.21%) had been subjected to oxygen supplementation and only three (15.79%) babies diagnosed with ROP had not been subjected to oxygen supplementation.

**Table 5:** Association between ROP and oxygen supplementation therapy ROP: retinopathy of prematurity.

Oxygen therapy	ROP		
	Present	Absent	Total
Yes	16	34	50
No	3	27	30
Total	19	61	80

The chi-square test of independence was not valid in this case, as the expected cell count was less than 5. Fisher’s exact test was performed to examine the association between ROP and oxygen supplementation. The association between these two variables was statistically significant. Fisher's exact test statistic was 0.0308 and the p-value was 0.0251.

The result was significant as the p-value was <0.05. The odds ratio was calculated to measure the strength of the association between ROP and oxygen supplementation, which was 4.2353, with a 95% confidence interval of 1.1172 to 16.056.

So, according to the analysis of the data, the odds of the presence of ROP in those who received oxygen supplementation was 4.2 times more than those who did not receive oxygen supplementation.

Zone distribution of ROP

Distributing the ROP babies into zones is important to assess the severity of ROP and decide upon the treatment for the ROP babies. Table 6 shows that out of the total number of subjects with ROP (19), 11 (57.89%) had ROP in zone III, six (31.58%) in zone II, and two (10.53%) in zone I.

**Table 6:** Zone distribution of ROP ROP: retinopathy of prematurity.

Zones	No. of subjects (out of 19)
I	2 (10.53%)
II	6 (31.58%)
III	11 (57.89%)

Staging in ROP-positive subjects

In our study, out of the 19 ROP-positive subjects, 10 (52.63%) subjects had stage II ROP, followed by five (26.32%) with stage I and four (21.05%) with stage III ROP, while no subjects were present with stage IV and stage V ROP.

Types of ROP in diagnosed cases

Table 7 shows that out of 19 ROP cases, six (32%) had type 1 ROP and 13 (68%) had type 2 ROP.

**Table 7:** Types of ROP in diagnosed cases ROP: retinopathy of prematurity.

Type	No. of cases	Percentage
1	6	32%
2	13	68%
Total	19	100%

Treatment of ROP-positive babies

**Figure 5:**
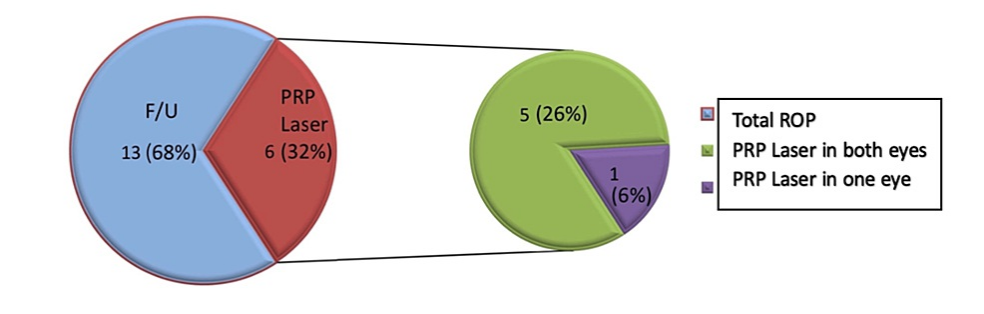
Treatment in ROP-positive babies ROP: retinopathy of prematurity; PRP: panretinal photocoagulation.

Figure 5 shows that out of 19 ROP cases, 13 (68%) received follow-up (F/U) care based on the severity of their disease and six (32%) were treated with PRP laser. Out of those who received laser treatment, five (26%) got laser treatment in both eyes whereas one (6%) got laser treatment in one eye only.

## Discussion

Today, there is an alarming increase in the incidence of ROP in developing countries, including India, which is referred to as the third epidemic of ROP. While the incidence of ROP is declining in the West due to advanced and well-monitored neonatal care, in India, it is on the rise due to high rates of premature births, sub-optimal neonatal care, and lack of stringent screening protocols. ROP can be due to multiple factors in low birth weight (LBW) babies before 34 weeks of gestation and the incidence increases with decreased gestational age at the time of birth. ROP is a potentially blinding disease and prevention is still the best strategy to avoid this blindness.

In this study, we took into consideration 160 eyes of 80 subjects who were delivered at or before 34 weeks of gestation, had birth weight of 2000 grams or less, those who received supplemental oxygen therapy or those infants who required NICU stay. Those who fulfilled one or more of the inclusion criteria were recruited prospectively. They were screened for the presence of ROP. We also studied the treatment provided to the diagnosed cases and documented the treatment outcomes.

The incidence of ROP in the present study was 24%. This is in concurrence with studies done by Patel et al. in Vadodara [6] between 2017 and 2018 with an incidence of 24.1% of the 284 neonates screened for ROP and by Thakre et al. in Maharashtra [7], where the incidence of ROP was found to be 27.73% of the 119 neonates screened. The incidence in the present study was found to be lower as compared to the study by Tekchandani et al. [8] conducted at a tertiary care institute in North India during 2013-2017 in which incidence was 32.3% and higher when compared with Singh et al. [9], who reported an incidence of 19% in their study conducted in Ajmer in 2016. The discrepancies in observation may be due to the varying sample size taken, differences in the study duration, and geographical areas where the studies were conducted.

In the present study of 160 eyes of 80 subjects, six infants (28%) were born at or before 28 weeks of gestation, nine (11.25%) at 29 weeks, 19 (23.75%) at 30 weeks, 16 (20%) at 32 weeks, four (5%) at 33 weeks, and seven (8.75%) at or after 34 weeks. Subjects with gestational age ranging from 28 weeks to 37 weeks were included. A total of 67% of the infants of 28 weeks developed ROP, followed by 56% of 29 weeks, 32% of 30 weeks, 19% of 31 weeks, 5% of 32 weeks, and no ROP cases in 33-37 weeks. This shows that the incidence of ROP decreases with increasing gestational age. The mean gestational age of screened infants was 31.01 ± 1.859 weeks, which is comparable to studies in India like Anudeep et al. [5] reporting a mean gestational age of 30.88 ± 2.383 weeks and Tekchandani et al. [8] with 31.3 ± 2.8 weeks. It was higher than that reported in the rest of the world like in the United States and Canada in a study by Quinn et al. [10], with a mean gestational age of 28 weeks, in Hong Kong in a study by Iu LP et al., with a mean gestational age of 27 weeks [11], in Taiwan in a study by Li ML et al. [12], with a mean gestational age of 27 weeks, and in Brazil in a study by Fortes Filho et al. [13], with a mean gestational age of 29 weeks. This is because the screening guidelines in these countries do not include more mature and heavier neonates due to their earlier observations of the incidence of ROP in only very premature and LBW neonates.

The mean birth weight of screened infants was 1618 ± 339.0 grams. It is comparable to other studies in India like a study by Dwivedi et al. in 2019 [14] where the mean birth weight was 1.63 ± 0.015 kg and a study by Thakre et al. in 2020 [7] where the mean birth weight was 1498 ± 317.2 g, but it is higher than studies from other parts of the world, recording mean birth weights as 1099 ± 259 g, 1285 ± 328 g, and 940 ± 1050 g, respectively [11-13]. Infants with weights ranging from 900 g to 2500 g were included in this study and around 36.8% of the diagnosed cases (out of 19) weighed >1500 g. These cases would have been missed had the study followed the UK (2022) screening guidelines, which recommend infants < 31 weeks of gestational age or <1501 grams birth weight be screened, or the American screening guidelines, which recommend screening for all infants with gestational age ≤ 30 weeks or those weighing ≤1500 grams at birth and at infants who have been exposed to risk factors of ROP weighing 1500-2000 g. As more and more mature and heavier babies are being affected by ROP in developing countries, it is important to include more mature and heavier babies in the screening criteria, or else cases in this group would go undiagnosed and might lead to visual impairment or even blindness.

The mean gestational age of ROP-positive babies was 29.58 ± 1.139 weeks (range = 28-32 weeks), which was lower as compared to babies without ROP, i.e., 31.46 ± 1.179 weeks (ranging from 28 to 37 weeks), and the difference between them was statistically significant (t value = 6.12, p < 0.0001). The mean birth weight of babies with ROP (1331.58 ± 238.532 g; range = 900 to 1700 g) was also lower as compared to babies without ROP (1706.56 ± 315.641 g; ranging from 1100 to 2500 g), and there was a statistically significant difference between them (t value = 4.76, p < 0.0001). This shows that the incidence of ROP increases with decreasing gestational age and birth weight, as seen in other studies [6,7]. Although an increased incidence of ROP in more mature and heavier babies is being noticed, prematurity and LBW remain the biggest risk factors.

The maximum number of subjects (36, 45%) were screened at ≤ one month of postnatal age, followed by 10 (12.5%) at two months, 10 (12.5%) at three months, nine (11.25%) at four months, and 15 (18.75%) at ≥ five months of age. The mean age at first screening was 2.76 ± 2.225 months (ranging from 0.5 to 10 months). According to the National Neonatology Forum, India (NNF) recommendation for screening in 2020 [15], the first screening of ROP should be performed at four weeks of postnatal age (can be preponed to two to three weeks of postnatal age in certain conditions). However in the present study, 55% of the babies were screened after one month (four weeks), and only 45% of the babies were timely screened; this suggests a lack of awareness and late referrals for screening and is one of the major factors responsible for the surge of visual impairment due to ROP.

Out of the 80 screened infants in our study, 12% were from upper-class families, 24% belonged to upper-middle-class families, 33% belonged to lower-middle-class families, 21% came from upper-lower-class families, and 10% of the subjects belonged to lower-class families. The maximum number of screened infants were from lower-middle-class families and the minimum number of them were from lower-class families. Screening rates of upper-class infants were also low in our study. However, since these families have better access to healthcare facilities, it can be assumed that they might be following up with a private practitioner. However, the low screening rates of lower-class family infants who do not have the means to get treated in private practice may be attributed to a lack of awareness about ROP, inadequate access to healthcare facilities, and also a lack of awareness about various schemes and programmes (like Rashtriya Bal Swasthya Karyakaram) by the government that are available to provide assistance in dealing with a disease like ROP and prevent childhood blindness. A study with a much larger sample size would be required to make any definitive statement.

In this study of 80 subjects, 37 (46%) were females and 43 (54%) were males. Out of the 19 diagnosed cases, 10 (52.6%) were males whereas nine (47.4%) were females, i.e., males and females were almost equally affected. A similar observation was made in a study by Suryawanshi et al. [16] showing seven (46.67%) male and eight (53.33%) female ROP cases. A male predominance was seen in a study by Le C et al. with 59% male and 41% female [2] and by Patel et al. with 55% male and 45% female ROP cases [6]. This may be due to the differences in the number of males and females screened. There was no statistically significant association between gender and the occurrence of ROP (p-value = 0.9108), which was in concurrence with other studies [6,7].

Prolonged oxygen therapy is a known risk factor for ROP. In the present study, out of 19 cases, 16 (84.21%) received oxygen supplementation and only three (15.79%) did not receive oxygen supplementation. A statistically significant association was found between oxygen supplementation and the development of ROP (p-value = 0.0251). This is comparable to various other studies showing oxygen therapy as a significant risk for ROP [6,7,9]. One of the reasons why the incidence of ROP is decreasing in developed countries is due to their strict guidelines and monitoring of oxygen therapy provided to infants, which is lacking in countries like India.

Out of 19 infants diagnosed with ROP, the maximum (11, 57.89%) had ROP in zone III, six (31.58%) infants had ROP in zone II, and the minimum number of infants (2, 10.53%) had ROP in zone I. Stage 1 ROP was seen in five (26.32%) cases, stage 2 in 10 (52.63%) cases, and stage 3 in four (21.05%) cases; none of the cases had stage 4 and stage 5 ROP. Our findings are rather similar to that of a study done by Le C et al. [2] in which ROP was most commonly seen in zone III (68%), followed by zone II (26%), and only one case in zone I; Hungi et al. [17] showing ROP most commonly in zone III (52.7%), followed by zone II (31.9%) and then zone I (15.4%); and study by Anudeep K et al. [5] in which 29.1% had stage 1 ROP, 37.5% had stage 2, and 33.3% had stage 3 of the 24 babies who developed ROP in their study of 65 subjects. The chances of inter-observer differences that might exist can be eliminated by developing a computer-based imaging technique.

Out of the diagnosed cases (19), six (32%) were type 1 ROP and 13 (68%) were type 2 ROP. If type 1 ROP is reported out of the total infants screened (80) in the present study, the incidence will be 7.5%. When compared with various studies for the incidence of type 1 ROP, the observation of the present study is somewhat comparable to studies done by Yu Y et al. with 5.9% of type 1 cases [18] of the 11463 neonates screened, Quinn GE et al. [10] with 6.1% of type 1 cases of the 7483 infants screened, Thakre et al. with 13 (10.9%) type 1 cases of the 119 neonates screened [7], and Goyal A et al. [19] reporting 9.95% type 1 cases out of 1648 eyes screened. However, a higher incidence was observed by Acevedo-Castellón et al. with 27.3% of type 1 cases out of the 132 neonates screened [20] and by Gaber et al. with 26.3% of type 1 cases out of 240 neonates screened [21]. Although the observation in the present study is similar to a lot of studies, as mentioned above, a much wider sample size is needed to confirm the findings. A higher incidence in the studies conducted in Brazil and Egypt, respectively, might be because these are developing countries [20,21].

In the present study, out of 19 ROP-positive babies, six (32%) required intervention with laser photocoagulation (PRP laser), five (26%) needed laser in both eyes whereas one (6%) needed it in only one eye. Out of the six babies who were treated with PRP laser, five needed only one laser sitting; however, one baby required a second laser sitting. As per the Early Treatment for Retinopathy of Prematurity (ETROP) study, babies with a high-risk pre-threshold (i.e., type 1) were given treatment. The rest of the 13 (68%) cases (type 2) were only followed up at weekly intervals and showed signs of spontaneous regression without intervention. Regression of the disease was observed in all those who received treatment with PRP laser. Other studies have also shown similar results following laser therapy [19]. Some studies have also shown the use of intravitreal anti-vascular endothelial growth factor injections [8] for the treatment of stage 3+ zone I, but it was not used in any of the cases in our study.

Limitations of the study

Each study comes with some limitations. In our study, the sample size was very small and along with that the study was conducted over a short duration of two months, so no definitive statements regarding trends that might exist in the general population could be made. Our study was restricted to one state of India and did not take into consideration the comparison with other or neighbouring states. We did not study the other risk factors, such as hypertensive disorders of pregnancy, maternal diabetes mellitus, maternal medication use, smoking, and premature rupture of membranes. There were also no cases recorded in stages 4 and 5, which are considered the most severe stages of ROP.

## Conclusions

The incidence of ROP in this study was 24%, with the data suggesting prematurity, low birth weight, and oxygen therapy as the most significant risk factors associated with the development of ROP. Early screening, timely intervention, and increasing awareness about the disease play a monumental role in reducing the burden of childhood blindness due to ROP in developing countries like India. For now, all infants ≤ 2000 g, born ≤ 34 weeks of gestation irrespective of the presence of risk factors should be screened for ROP and strict guidelines for oxygen therapy should be implemented. Additionally, mobile ophthalmic units should be used to conduct mass screening of infants in out-of-reach areas and slums free of cost, and further management of the cases should also be free or at minimum fees.
